# Vitamin D status among pregnant women of North Macedonia: Assessing deficiency rates and associated risks factors

**DOI:** 10.5937/jomb0-55601

**Published:** 2025-09-05

**Authors:** Boshku Aleksandra Atanasova, Vasko Aleksovski, Krstevska Slagana Simeonova, Nikolovska Elena Gjorgievska, Igor Samardjiski, Markova Ana Daneva

**Affiliations:** 1 University Clinic of Gynecology and Obstetrics, Faculty of Medicine, Ss. Cyril and Methodius University, Skopje, North Macedonia; 2 University Clinic of Neurology, Faculty of Medicine, Ss. Cyril and Methodius University Skopje, North Macedonia

**Keywords:** 25(OH) vitamin D, woman, pregnancy, deficiency, supplementation, 25(OH) vitamin D, žena, trudnoća, deficit, suplementacija

## Abstract

**Background:**

Vitamin D is an essential vitamin that plays a key role in maintaining overall health. During pregnancy, the demand for vitamin D increases to support the growing needs of the fetus. Vitamin D deficiency is highly prevalent in pregnant women worldwide. Our study aimed to evaluate the vitamin D deficiency rate among pregnant women in the Republic of North Macedonia, along with influencing factors.

**Methods:**

We conducted a prospective study of randomly selected pregnant women with different body mass indexes among vitamin supplementation users and non-users in two different seasons.

**Results:**

A total of 309 pregnant women aged > 18 years were recruited from June 2022 to April 2023, with an average vitamin D concentration of 38.9 (36.6-40.2) nmol/L. During winter, 80.8 % of pregnant women had vitamin D deficiency.

**Conclusions:**

Even though more than 77.3 % of pregnant women consume multivitamins containing vitamin D, vitamin D deficiency is highly prevalent among pregnant women, especially among obese pregnant women and during the winter months.

## Abstract (sr)

**Uvod:**

Vitamin D je esencijalni vitamin koji igra ključnu ulogu u održavanju opšteg zdravlja. Tokom trudnoće, potreba za vitaminom D se povećava kako bi se zadovoljile rastuće potrebe fetusa. Deficit vitamina D je veoma rasprostranjen među trudnicama širom sveta. Cilj ove studije je bio da se proceni stopa deficita vitamina D kod trudnica u Republici Severnoj Makedoniji, kao i faktori koji na njega utiču.

**Metode:**

Sprovedena je prospektivna studija na nasumično odabranim trudnicama sa različitim indeksom telesne mase, u dva različita godišnja doba, među korisnicama suplemenata vitamina D i onima koje to nisu.

**Rezultati:**

U istraživanje je uključeno ukupno 309 trudnica starijih od 18 godina, u periodu od juna 2022. do aprila 2023. godine, sa prosečnom koncentracijom vitamina D od 38,9 (36,6-40,2) nmol/L. Tokom zimskog perioda, deficit vitamina D je utvrđen kod 80,8 % trudnica.

**Zaključak:**

Uprkos tome što više od 77,3 % trudnica konzumira multivitamine koji sadrže vitamin D, deficit vitamina D je i dalje veoma prisutan, posebno kod gojaznih trudnica i tokom zimskih meseci.

## Introduction

Vitamin D is a vital nutrient that plays a crucial role in supporting overall health and well-being. Often referred to as the »sunshine vitamin,« it is unique because the body can produce it when the skin is exposed to sunlight. Despite its importance, many people worldwide suffer from vitamin D deficiency, leading to various health issues ranging from weakened bones to compromised immune systems [1].

### Importance of vitamin D during pregnancy

Vitamin D plays a multifaceted role during pregnancy. It is crucial for regulating calcium and phosphorus, which are vital elements for the development of the fetal skeleton, influences bone health and immune function, and possibly reduces the risk of various diseases later in life [2]. The fetus relies on maternal vitamin D deposits as it cannot synthesise vitamin D during gestation. Adequate maternal vitamin D levels have been associated with improved birth outcomes, including higher birth weight and a reduced risk of preterm birth [3]. Vitamin D deficiency in pregnant women is linked to an increased risk of gestational diabetes and hypertensive disorders, such as pre-eclampsia, and higher odds of small for gestational age (SGA) or low birth weight babies (LBW) [4]. Studies indicate that a significant proportion of pregnant women in various European countries do not meet the recommended vitamin D levels, particularly during the winter months when sunlight is limited [5].

### Current guidelines for vitamin D intake

Determination of the serum level of 25 – hydroxyvitamin D [25(OH)] is the most broadly used method for evaluating vitamin D status [2] [6]. However, there is no consensus regarding vitamin D screening or optimal vitamin D levels during pregnancy. The Institute of Medicine (IOM) defines serum 25(OH)D levels lower than 30 nmol/L as insufficient and levels greater than 50 nmol/L as sufficient [7], while the Endocrinology Society advocates a threshold of 75 nmol/L [2].

Different societies have different recommendations regarding vitamin D supplementation. WHO 2020 guidelines recommend no need for supplementation in populations with normal levels. However, in cases where it is challenging to maintain adequate vitamin D levels through natural sources alone, the WHO recommends 200 IU Vitamin D supplementation for pregnant women with suspected deficiency [8]. Current European guidelines recommend a daily intake of 10–20 mg vitamin D for pregnant women, depending on the geographical location and individual risk factors [9] [10]. These recommendations aim to ensure adequate serum levels of 25-OH vitamin D, essential for maternal and fetal health. Despite these guidelines, compliance and awareness remain issues that underscore the need for public health initiatives to promote adequate vitamin D intake in pregnant women.

Healthcare providers across Europe have implemented various measures to improve vitamin D levels during pregnancy. These include educational campaigns for pregnant women regarding the importance of vitamin D and sunlight exposure, guidelines on dietary modifications to include vitamin D-rich foods and recommendations for vitamin D supplementation, especially during the winter months [11]. Furthermore, prenatal care providers increasingly emphasise the importance of adequate maternal vitamin D levels throughout pregnancy to ensure optimal health outcomes for both the mother and developing fetus.

### Vitamin D deficiency in the Balkans

Despite living in the Northern Hemisphere and having abundant sunlight, reports have indicated that vitamin D deficiency is widespread among people living in the Balkan region, including pregnant women. For example, a study conducted in Serbia reported that over 47% of pregnant women had insufficient vitamin D levels [12], whereas in Bulgaria, a study indicated that approximately 31.98% of pregnant women were deficient in vitamin D [13].

Similarly, published data from Greece have indicated that despite enjoying a high exposure to sunlight throughout the year, the population is an example of the Mediterranean paradox, as it suffers from Vitamin D deficiency [14] [15].

The prevalence of deficiency can be attributed to several factors, including limited duration of sun exposure, lifestyle factors, and dietary habits. In many Balkan countries, traditional diets lack sufficient sources of vitamin D, such as fatty fish and fortified foods.

Macedonian women differ somewhat from European women regarding sun exposure according to geographic latitude, climate, and attitude towards sunbathing. There is no recommendation for the routine screening of vitamin D levels in pregnant women in North Macedonia. However, the recommendations for vitamin D intake and serum levels for pregnant women in North Macedonia generally align with the international guidelines. Nevertheless, research evaluating the effectiveness of vitamin D supplementation during pregnancy is insufficient, and no study has reported the frequency of vitamin D deficiency in pregnant women during the first trimester.

In contrast to many other countries that have implemented their own guidelines and recommendations for optimal vitamin D levels during pregnancy and have introduced strategies to prevent vitamin D deficiency, North Macedonia lacks national guidelines dedicated solely to vitamin D levels in pregnant women.

Our study aimed to define the mean levels of 25(OH)D in pregnant women in North Macedonia, evaluate the prevalence of moderate-to-severe deficiency, and determine the associations between levels and age, body mass index (BMI), seasons, and vitamin D.

## Materials and methods

This was a single-centre population-based cross-sectional study. This study included 309 pregnant women aged > 18 who underwent routine prenatal combined noninvasive screening procedures during pregnancy at the University Clinic for Gynecology and Obstetrics, Skopje, North Macedonia. This study was conducted between June 2022 and March 2023. This period was chosen to predict the effect of sun exposure during and after the summer months and during minimal exposure to sunlight, owing to the shortage of days and weather conditions in winter. All participants provided informed consent for inclusion in this study. This study was conducted following the principles of the Declaration of Helsinki. The inclusion criterion was healthy Caucasian women with singleton pregnancies. The exclusion criteria were age < 18 years, twin pregnancy, congenital anomalies of the fetus, previously diagnosed maternal diseases, eating disorders, diabetes mellitus, short bowel syndrome, celiac disease, Crohn’s disease, ulcerative colitis, a history of gastrointestinal surgery, and HIV/AIDS infection.

### Data collection

A multiple-choice questionnaire was developed to collect demographic data (age, education, marital status, and occupation). Participants completed a questionnaire regarding vitamin use during pregnancy. Detailed information on the supplements used by the participants was provided (type, brand, dosage per day, and duration of the supplementation). Daily vitamin D intake was assessed according to the reported consumption and manufacturer’s information on vitamin D content in a particular preparation. Trained personnel (nurses with higher education, medical students, or residents) conducted interviews.

We recorded weight changes during the first trimester of pregnancy by calculating body mass index (BMI, kg/m^2^) before pregnancy and at the time of data collection. BMI was classified as per the WHO criteria and is as follows: underweight (<18.5 kg/m^2^), normal weight (18.5–24.9 kg/m^2^), overweight (25.0–29.9 kg/m^2^), and obese (>30.0 kg/m^2^) [16].

### Measurement of 25(OH)D level from blood samples and cut-off points for vitamin D

Maternal blood samples were collected during standard testing. Serological tests were performed on a blood sample collected in an SST tube (Clot Activator and Gel, BD, USA), which was allowed to stand for 15 min and then centrifuged (3500 rpm for 15 min).

For the assessment of vitamin D Levels in each patient, we considered the serum levels of 25(OH) vitamin D (ng/mL), which were routinely determined by a one-step delayed-action immunoassay using direct chemiluminescence with advanced acridinium ester technology (CLIA) using relevant commercial kits (ADVIA Centaur Vitamin D Total, Siemens) on the Advia Centaur XPT system. According to the manufacturer, the functional sensitivity of the assay is 10.5 nmol/L (4.2 ng/mL), and the linearity through the measurement range is between 10.5 nmol/L and 375 nmol/L (4.2 ng/mL to 150 ng/mL). The limit of blank (LoB), the limit of detection (LoD) and the limit of quantitation (LoQ) is 4.3 nmol/L (1.7 ng/mL), 8.0 nmol/L (3.20 ng/mL) and 10.5 nmol/L (4.2 ng/mL), respectively. The ADVIA Centaur Vit D assay is standardised using internal standards that are traceable to IDLC/MS/MS 25(OH) vitamin D RMP. ID-LC/MS/MS was traceable to the National Institute of Standards and Technology Standard Reference Material (2972). Three levels of Bio-Rad Liphochek control were used for verification, as well as a monthly Riquas immunology external control.

For data analysis, the recommendations from the IOM were used to categorise vitamin D status by serum 25(OH) vitamin D levels as follows: severe deficiency, <25 nmol/L; deficiency (<50 nmol/L) insufficiency, (50–74 nmol/L); and adequacy (>75 nmol/L) [17].

### Statistical analysis

SPSS 20.0 version (SPSS Inc., Chicago, IL, USA) was used for statistical analysis. Descriptive statistical data were expressed as means, standard deviations, and minimum, maximum, median, and categorical variables as frequencies and percentages. The Kolmo gorov-Smirnov and Shapiro-Wilk tests were used to assess the assumption of normality. Numerical variables are presented as mean±standard deviation and median (25^th^–75^th^ percentile). Categorical variables are summarised as counts (percentages). An independent-sample t-test for numerical variables determined differences between groups with a normal distribution, and the Mann-Whitney U test for numerical variables without a normal distribution. Relationships between categorical variables were evaluated using the chi-square test, Fisher’s exact test, and Fisher-Freeman-Halton exact test. Statistical significance was set at p<0.05.

## Results

Of the 350 patients in this study, 309 met the specified inclusion criteria and were included in the final analysis. The general demographic and clinical characteristics of the pregnant women’s cohort are presented in Table 1. The statistical evaluation of data from 309 pregnant women has shown that our group’s mean age was 28.82±4.8 years, median 29 years (17.0–44.0) and had a median BMI of 31.06±2.37 kg/m^2^. Pregnant women had a lower pre-pregnancy body weight; however, 72.1% had a body mass index higher than 25 kg/m^2^ during pregnancy.

**Table 1:** Baseline characteristic of first-trimester pregnant women. Notes: SD = standard deviation; BMI = body mass index

Variable		1^st^-trimester <br>pregnancies
		(18–44 years old)
		*N*=309
Age <br>(mean ± SD)		28.7±4.41
Gestation week <br>(mean ± SD)		13.3±7
Education (%)	primary school	45 (14.6%)
	high school	142 (45.9%)
	higher education	122 (39.5%)
Body weight (kg)		84±23.7
BMI kg/m^2^ <br>(mean ± SD)		31.06±8.1
BMI kg/m^2^	<25	87 (27.9%)
	≥25	223 (72.1%)
Smoking status <br>(%)	current smoker	55 (17.6%)
	ex-/non-smoker	254 (82.4%)
Physical activity <br>(%)	low level	96 (31.2%)
	moderate level	99 (32%)
	high level	114 (6.8%)
Vitamin D supplement us<br>	users	243 (78.6%)
	non-users	66 (21.4%)
Season (%)	summer <br>(June–October)	137(44.3%)
	winter <br>(November–April)	172 (55.7%)

For the whole group (309 pregnant women), the mean 25(OH) D level was 38.88±14.4 nmol/L; the median was 38.4 nmol/L (10.7–92.3 nmol/L), which corresponds with insufficient levels (Table 2). Twenty-two per cent of women were overweight, with a BMI between 25–30 kg/m^2^ and a mean vitamin D concentration of 37.0±12.3 nmol/L. Only 29.5% of the participating women had a normal body mass index at the time of blood collection, and most had sufficient vitamin D levels (45.2±16.6 nmol/L). There was a statistically significant difference in the mean vitamin D concentration among the three groups according to BMI (p<0.001), with the highest levels observed in pregnant women with a normal BMI. We evaluated 25(OH) vitamin D concentrations during the summer months (44.3%) and during the winter months in 55.7% of the participating pregnant women. During the summer period, 52.8% of the women had sufficient levels of vitamin D. Most of the deficiencies were recorded during the winter (60.2 Plasma vitamin D concentrations were subdivided into three categories based on the Institute of Medicine (IOM) recommendations for vitamin D categorisation: sufficient (>75 nmol/L), insufficient (30– 50 nmol/L), and deficient (<30 nmol/L). According to this classification, 53.1% of women with BMI >30, 25.5% of women with BMI 25–30, and 21.4% of pregnant women with BMI <25 had vitamin D deficiency (< 30 nmol/L). Vitamin D insufficiency levels were found in 31.2% of normal-weight pregnant women, 24.1% of overweight pregnant women, and 47.% of obese pregnant women. Sufficient levels of vitamin D were found in 30.0% of normal-weight, 22.9% of overweight, and 47.1% of obese pregnant women. Vitamin D concentration was associated with season, and significant differences were found between winter and summer (Table 3). Namely, during the summer months, 52.8% of pregnant women had sufficient levels of vitamin D versus 47.1% during winter (p<0.05); 43.3% had insufficient levels during summer and 56.7% during winter (p<0.05); 39.8% had deficient levels during summer months, and 60.2% during winter months (p<0.05). Most of our pregnant population (n=243, 78.6%) received some form of prenatal multivitamin supplementation containing approximately 400 IU/day of vitamin D. We did not observe any differences in Vitamin D concentrations between women who were users of vitamin D supplements and those who did not use vitamin D as a supplement.

**Table 2:** The prevalence of maternal vitamin D according to IOM classification, prenatal supplements and sun exposure during pregnancy. Notes: SD = standard deviation; BMI = body mass index; * Statistically significant p-value. Values are presented as n (%) or mean ± SD

	All (n=309)	Vitamin D <br>Sufficient <br>(>50 nmol/L); <br>n=70 (22.7%)	Vitamin D <br>Insufficient <br>(30–50 nmol/L); <br>n=141 (45.6%)	Vitamin D <br>Deficient <br>(<30 nmol/L); <br>n=98 (31.7%)	*p* value
Age*	28.7 (4.4)	28.9 (4.5)	28.6 (4.5)	28.6 (4.0)	**0.701**
**BMI (Body Mass Index)**					
<25	87 (27.8%)	21 (30.0%)	44 (31.2%)	21 (21.4%)	**0.141**
25–30	75 (24.3%)	16 (22.9%)	34 (24.1%)	25 (25.5%)	
>30	148 (47.9%)	33 (47.1%)	63 (44.7%)	52 (53.1%)	
**Season**					
Winter	172 (55.7%)	33 (47.1%)	80 (56.7%)	59 (60.2%)	0.027*
Summer	137 (44.3%)	37 (52.8%)	61 (43.3%)	39 (39.8%)	
Vitamin D Supplement					
Yes	243 (78.6%)	54 (77.1%)	111 (21.3%)	78 (79.6%)	**0.366**
No	66 (21.4%)	16 (22.9%)	30 (78.7%)	20 (20.4%)	

**Table 3:** Serum vitamin D concentration in pregnant women according to BMI, season, and vitamin D supplementation. Notes: BMI = body mass index; * p-values obtained using the Kruskal-Wallis test; **p-values obtained using Mann- Whitney U test; Statistically significant p-value: p<0.05.

Category	Vitamin D (nmol/L), <br>Mean (95% CI)	p-value
**All participants <br>n=309**	38.9 (36.6–40.2)	-
**BMI (Body Mass <br>Index)**		0.001*
<25	45.5 (41.75–48.15)	-
25–30	37.9 (34.9–40.9)	-
>30	35.4 (33.7–37.4)	-
**Season**		0.001*
Winter	33.4 (31.0–35.3)	-
Summer	46.1 (43.6–48.6)	-
**Vitamin D <br>Supplementation**		0.237**
Yes	39.3 (37.4–41.2)	-
No	37.7 (34.3–41.1)	-

## Discussion

This study investigated the prevalence of hypovitaminosis D in a healthy pregnant woman (28.8±4.4 years) at a tertiary university centre in Skopje, North Macedonia, Europe, with an average gestational week of 13.0±6 days. We examined a healthy pregnant woman without preexisting comorbidities, which could be a reason for vitamin D deficiency. The results of our study highlight that the median serum 25(OH)D level found in our study (38.9 nmol/L) is far below the average of some North European studies of pregnant women (85.0 nmol/L, Latvia), which is significantly lower than the median found in a Swedish study (64.0 nmol/L) [18].

Studies have shown that individuals living at higher altitudes often have lower serum levels of vitamin D, primarily because of the reduced intensity of ultraviolet B (UVB) radiation, which is necessary for the skin to produce vitamin D [2]. For instance, in Serbia, where large areas are located at higher elevations, this geographic factor must be considered to address vitamin D deficiency, especially among pregnant women at greater risk of adverse pregnancy outcomes due to insufficient vitamin D levels [12]. A high prevalence of vitamin D deficiency was observed in 47.0% of all mothers and 77.0% of all infants in Balkan County.

Similarly, research in Bulgaria has shown that vitamin D deficiency is prevalent in pregnant women, with significant variations between regions depending on latitude and lifestyle factors [19]. In our study, we have observed a prevalence of vitamin D deficiency of 31.4%, and insufficiency was observed in 50.0% of pregnant women, which ranks North Macedonia among countries with high prevalence of vitamin D in pregnant population in Europe [15] [18] [19].

North Macedonia is a mid-latitude country (latitude 41.6° North) but has minimal epidemiological data on vitamin D status among the population. As in many countries, Macedonia’s geographical location influences its residents’ health, particularly regarding vitamin D production. The country experiences four distinct seasons: long winter, cold winter, and hot summer. Latitude is crucial in determining the sunlight a region receives throughout the year. For countries farther from the equator, like North Macedonia, sunlight exposure can vary significantly by season. During winter, sunlight is less direct, reducing the skin’s vitamin D synthesis. We observed that seasonal and lifestyle-related factors modify 25(OH)D levels during pregnancy. The primary source of vitamin D is dermal synthesis induced by UVB [20]. This study investigated seasonal variation in vitamin D status among pregnant women. The observation period was divided into winter (November–April) and summer (June–October). During winter, the observed prevalence of vitamin D deficiency was 34.3%, 46.5% was insufficient, and only 19.2% had sufficient levels.

During summer, deficiency was observed in 28.0 % of pregnant women. Thus, there was a statistically significant difference between mean values of vitamin D levels during summer and winter. However, according to IOM, the mean value during summer (46±14.7) did not reach recommended levels more than 50.0 nmol/l. There was a positive correlation between vitamin D concentration and season. In multiple regression analysis, only the duration of sunbathing remained a significant factor influencing vitamin D levels. The lower seasonal variability in vitamin D levels among the pregnant population might be related to their sun-avoiding behaviour and use of sunscreens as protective factors against melanoma.

Obesity is a known risk factor for vitamin D deficiency [21]. As a fat-soluble vitamin, vitamin D is mainly stored in adipose tissue. In individuals with a higher fat mass, more vitamin D is sequestered, reducing its bioavailability in the circulation. Obesity is sometimes associated with reduced physical activity and less time spent outdoors, which can decrease exposure to sunlight, a critical factor for endogenous vitamin D synthesis [22]. In our sample, 72.1% of pregnant women were overweight or obese, which may have affected the high prevalence of vitamin D deficiency/insufficiency. Peak blood concentrations of vitamin D significantly differed between obese and non-obese pregnant women. The reduced bioavailability of vitamin D3 synthesised in the skin owing to obesity emphasises the biological mechanisms underlying this association. In pregnant women with obesity, the increased fat mass acts as a sink, trapping vitamin D and reducing its availability in circulation. This reduction limits their active roles in calcium metabolism, immune function, and other vital processes in mothers and children, thereby adding value to the already risky environment caused by obesity.

There are numerous reasons for the decline in vitamin D levels in pregnant women, such as increased BMI, increased use of sun blockers, dietary habits with minimal intake of oily fish, and products containing vitamin D, such as milk and dairy products; thus, it is closely related to the socioeconomic status of the woman as well as the level of education.

Vitamin D supplementation, particularly multivitamins, has garnered attention because of its potential efficacy in pregnant women. Regular use of multivitamins has been suggested as one of the mechanisms for maintaining adequate vitamin D. Unfortunately, an increasing number of studies have indicated that increased use of multivitamins in young and older populations is not associated with an appropriate increase in serum 25(OH) vitamin D concentrations.

Some recommendations for vitamin D supplements state that doses should not differ from those of non-pregnant women and that 15 mg/day (600IU) is sufficient to maintain a stable level. However, these guidelines do not recommend vitamin D levels in the mother’s blood during pregnancy [9].

However, the high global rate of hypovitaminosis D is alarming. Several authors have revised the standard recommendations for vitamin D supplementation and increased doses up to 1000 IU per day in both the general population and pregnant women, especially those at risk for hypovitaminosis D [5] [18] [23] [24].

In our study, most pregnant women (78.6%) took some form of pregnancy multivitamins containing 200–400 IU vitamin D3. The main concentration of serum vitamin D did not differ significantly between users and non-users of multivitamins. Measured serum vitamin D levels reflect the insufficient vitamin D intake of included women. According to our results, 22.7% had reached more than 50 nmol/L, and 81.0% did not reach adequate (50 nmol/L) vitamin D serum levels. The present results support previous studies that reported that 400 IU daily may not be acceptable to maintain sufficient vitamin D levels in pregnant women [25] [26]. We also support the opinion of studies that suggested 1000IU, 2000IU, and 4000IU doses of vitamin D, particularly among pregnant women living in higher latitudes, winter periods, and high-risk pregnancies [18] [23] [24] [27]. However, the multifactorial nature of vitamin D deficiency suggests that, while multivitamins can aid in improving vitamin D status, they may not be a comprehensive solution for all pregnant women. The lack of an evaluation of dietary factors and calcium intake can all be considered a limitation of this study. As a single-centre study, our work has inherent strengths and limitations. The unified approach to diagnosis and treatment by our consistent staff enhances internal validity. Our hospital serves both urban and rural areas across all socioeconomic groups in R. Macedonia, but selection bias may persist. Despite these limitations, our findings could particularly benefit high-risk groups, including socioeconomically vulnerable pregnant women who face elevated risks of adverse maternal and neonatal outcomes. Given that healthcare workers and pregnant women often follow official guidelines, our results could inform more precise vitamin D supplementation recommendations, potentially benefiting the broader pregnant population.

## Conclusion

Maintaining adequate vitamin D levels during pregnancy and lactation is of particular importance. Our study found that our pregnant population was severely deficient in vitamin D, with only 22.7% of pregnant women in our cohort having adequate levels. One of the presumed reasons may be dietary habits, namely, minimal intake of foods containing vitamin D, lack of vitamin D fortification of foods, and low subcutaneous production, especially in winter, due to minimal UVB exposure at 41.6°N latitude. In this study, multivitamins did not significantly affect vitamin D levels. The use of multivitamins containing vitamin D is associated with modest improvements in serum vitamin D levels among pregnant women, and their effectiveness can vary depending on individual circumstances, including BMI, lifestyle, and dietary habits. Current evidence suggests that higher doses of vitamin D supplementation may be necessary to achieve optimal vitamin D levels during pregnancy, especially in populations at risk of vitamin D deficiency. This underscores the influence of sociodemographic, lifestyle, and environmental factors on the vitamin D status in pregnant women. These findings provide valuable insights into changes in preventive measures towards promoting healthy lifestyles and adequate maternal nutrition. Future research should focus on determining the most effective doses and forms of vitamin D supplementation to ensure adequate maternal and fetal health.

## Dodatak

### Conflict of interest statement

All the authors declare that they have no conflict of interest in this work.
